# Different amount of training affects body composition and performance in High-Intensity Functional Training participants

**DOI:** 10.1371/journal.pone.0237887

**Published:** 2020-08-20

**Authors:** Valentina Cavedon, Chiara Milanese, Alessandro Marchi, Carlo Zancanaro

**Affiliations:** Laboratory of Anthropometry and Body Composition, Department of Neurosciences, Biomedicine and Movement Sciences, University of Verona, Verona, Italy; University of Rome, ITALY

## Abstract

The effects of High-Intensity Functional Training (HIFT) on body composition and the relationship of the latter with performance are not well defined. In this work we investigated, by means of Dual-energy X-ray Absorptiometry, the relative proportions of fat-, lean soft tissue-, and mineral mass in CrossFit^®^ (CF, a popular mode of HIFT) participants (n = 24; age, 28.2 ± 3.39 y; BMI, 25.3 ± 2.04 kg/m^2^) with at least 1 year of CF training experience and weekly amount of training > 10 h/w (n = 13; Higher Training, HT) or < 10 h/w (n = 11; Lower Training, LT) as well as age- matched and BMI-matched physically active controls (CHT, CLT). Performance was assessed in the “Fran” workout. Data were analyzed by one-way or repeated measures ANOVA where needed. Association between variables was assessed with the Pearson’s correlation coefficient r. Partial correlation was used where needed. Results showed that HT performed better than LT in the “Fran” (P < 0.001) and they had higher whole-body bone mineral density (P = 0.026) and higher lean soft mass (P = 0.002), and borderline lower percent fat mass (P = 0.050). The main difference between CF participants (HT, LT) and their respective controls (CHT, CLT) was a lower adiposity in the former. In CF participants, performance positively correlated with appendicular lean soft tissue mass (P = 0.030). It can be concluded that, in CF participants, a higher amount of weekly training improves most notably lean body mass and increases performance in association with increased skeletal muscle mass. CF participation is especially effective in reducing fat mass vs. age- and BMI-matched physically active controls.

## Introduction

Over the last several years, High-Intensity Functional Training (HIFT; [1]) has been constantly gaining popularity, possibly due to a greater degree of enjoyment for the participants and shorter training duration vs. conventional training methods, while improving fitness and overall health [2,3]. CrossFit^®^ (CF; CrossFit, Inc., Washington, DC, USA) is a mode of HIFT, which has gained a very large number of participants worldwide, with over 400,000 participants in the 2018 CF Open [4]. CF encompasses many types of functional movement patterns over a short duration (i.e., less than 30 minutes), with a high-volume, and high-intensity exercise program [5] within a single exercise session, called “Workout of the Day” (WOD). A typical WOD may involve a combination of cardiovascular activities (e.g., running, rowing and cycling), weightlifting/powerlifting exercises (e.g., clean and jerk, squat, deadlift, push press, bench press, and power clean), and elements of gymnastic exercises (e.g., handstand and ring exercises) performed in a timed and/or circuit format with little to no rest periods [6–8]. WODs can be performed with the objective of the best time or can be performed in the “as many rounds as possible” style using varying time domains, ranging from 10 to 20 minutes. Although the structure of each WOD will vary between CF affiliated organizations, each session typically lasts 1 hour and consists of a specific warm-up followed by a program of strength or a conditioning workout for 10–30 minutes and finally a cool down and/or mobility work.

Despite its growing popularity, little research has been carried out on CF. A recent systematic review and meta-analysis aimed at an overview of CF’s outcomes [9] only included 31 papers in the systematic review. In particular, little investigation has been carried out of body composition in CF participants. In the above quoted review paper [9] only four works deal with the issue, among others, of body composition in CF. Interestingly enough, the results of the meta-analysis revealed no “significant effect of CF training changes in body mass index, relative body fat, fat mass, lean body mass, and waist circumference” [9]. Even less information is available in the literature on the bone status in CF participants [10]. Moreover, to the best of our knowledge there is no information available in the literature on possible associations between body composition, amount of training and performance in CF. It is apparent that research is needed to evaluate the effects of CF participation on body composition in more depth, as well as the relationship of the latter with performance. To contribute towards filling this gap of knowledge, the present cross-sectional study of male athletes with at least 1 year of CF training experience was designed with a twofold aim. The first aim was to assess the effect of different amounts of CF training on body composition and performance in male CF participants as well as the correlation between body composition components and performance. The second aim was to assess whether body composition of CF participants is different from that of controls, matched by age- and BMI. Body composition was evaluated using the three-compartment model yielded by dual-energy X-ray absorptiometry (DXA) thereby allowing insights into the CF-related pattern of fat mass (FM), lean soft tissue mass (LSTM), and areal bone mineral density (aBMD) at the whole-body (WB) and regional level. CF performance was evaluated using a typical WOD, known as “Fran”.

## Materials and methods

### Participants

The study conformed to the Declaration of Helsinki (revised 2013) and was approved by the Verona University IRB. All participants gave written informed consent.

Male participants (> 18 years old) were recruited by putting up information sheets at the local CF gyms. Inclusion criteria were: 1) to be active members of a CF facility (i.e., attending at least one class per week) and have at least 1 year of CF training experience; 2) not taking any prescribed or over-the-counter medication for two weeks preceding the study; 3) to be in possession of a certificate of suitability for competitive sports issued by a specialist in Sports Medicine; 4) to pass the Physical Activity Readiness Questionnaire (PAR-Q; Canadian Society for Exercise Physiology) [11]. Exclusion criteria were: 1) the presence of any current cardiovascular, respiratory, or musculoskeletal concerns that would limit the participant’s ability to perform high-intensity exercise; 2) the presence of injury in the previous six months.

Overall, twenty-four CF athletes (age, 28.2 ± 3.39 y; body mass, 78.6 ± 8.00 kg; stature, 176.4 ± 4.36 cm; body mass index [BMI], 25.3 ± 2.04 kg/m^2^) from two CF training facilities fulfilled all inclusion and exclusion criteria and volunteered in this study. The two CF training facilities were supervised by the same group of coaches, so all participants followed an identical program of workouts. Data collection took place on two separate sessions, which were 48h apart. The first session consisted of administering and explaining the informed consent form to the participants and then the PAR-Q as well as filling out a written questionnaire including demographic data, total weekly hours of CF training, CF experience (i.e., years of CF participation) and injury history before or while practicing CF. Then, anthropometry and body composition analysis were carried out. In the second session, which took place at the participant’s affiliated gym, participants performed the “Fran” CF workout. All participants were asked not to significantly change their habits in terms of food intake and physical exercise in the week preceding the workout as well as to abstain from caffeine and alcohol intake 12 hours prior. Moreover, all participants were asked to complete the workout after no less than 48 hours of recovery time from a previous workout.

The study was carried out in winter (December-February).

### Anthropometry and body composition analysis

Body mass was taken at the nearest 0.1 kg with an electronic scale (Tanita electronic scale BWB-800 MA); stature was measured with a Harpenden stadiometer (Holtain Ltd., Crymych, Pembs. UK) at the nearest 0.01 m; BMI was calculated as weight (kg) / height (m^2^).

WB- and regional body composition (FM, %FM, LSTM, and aBMD) was evaluated by means of DXA using a total body scanner (QDR Explorer W, Hologic, MA, USA; fan-bean technology, software for Windows XP version 12.6.1) according to the manufacturer’s procedures. Quality control was carried out daily against a reference phantom supplied by the manufacturer to avoid possible baseline drift. In our lab, the in vivo short-term precision for total-body DXA measurements, calculated by repeated scanning of subjects according to the convention of the International Society for Clinical Densitometry (http://www.iscd.org/), is 2.3%, 2.8%, 0.5%, and 0.9% for FM, %FM, LSTM, and aBMD respectively. All analysis was performed by the same operator to ensure consistency. Scans were performed in late morning, in a post-absorptive state. Participants were asked to refrain from vigorous exercise and not to consume alcohol for at least 24 h before they arrived at the laboratory. Participants wore light-weight clothing with no metal or reflective material and removed all metal accessories. Velcro restraints were applied around participants’ ankles to ensure there was no movement during the scan.

For the standard regional body composition estimations, Hologic software readings divided the body into trunk, entire arm (left and right), entire leg (left and right), and head. In addition to the standard DXA output, the sum of arms and legs FM and LSTM were calculated (Appendicular FM and Appendicular LSTM, respectively). Appendicular LSTM is a proxy of skeletal muscle mass [12]. The Arms (average of right and left arm), Legs (average of right and left leg), and Appendicular (average of right arm and leg, and left arm and leg) aBMD was calculated as well. FM index (FMI) and LSTM index (LSTMI) were respectively calculated by dividing DXA-derived FM (in kg) and LSTM (in kg) by squared height (in meters).

### CF workout

The “Fran” workout is a fast WOD that has more anaerobic components and represents one of the CF benchmark workouts which is commonly used as a dependent variable for the analysis of performance [4]. Before the “Fran”, each participant performed a standardized warm-up consisting of running around the gym, multiple joint movements, skipping jumps, push-ups, knee bends, arms swings, etc. The “Fran” workout involved performing barbell thrusters and pull-ups following a 21-15-9 repetition scheme. The “Fran” scheme consisted in completing 21 thrusters and 21 pull-ups, then 15 thrusters and 15 pull-ups, then 9 thrusters and 9 pull-ups, as fast as possible. Thrusters (a front squat to push press) were performed with 43.2 kg and variations of pull-ups (including butterfly and kipping) were encouraged. The time to complete all repetitions was recorded and expressed in seconds. The CF workout was both supervised and scored by a CF Level 1 trainer to ensure the movement and workout standards were met. All participants performed the prescribed exercises and workouts with no modifications or scaling.

### Capillary blood analysis and heart rate measurement

Capillary blood lactate was evaluated with a portable analyzer (Lactate Plus; Nova Biomedical Waltham, MA, USA) on a drop of blood collected from the index finger of the participant’s hand. Capillary blood glucose levels were measured with a portable monitor (Terumo Finetouch; Tokyo, Japan), on a drop of blood collected in the same way. All measurements were taken at three different times: at rest, 5 min before the start of the performance and after the participant had been lying on the ground for 15 min (Baseline), immediately after the end of the “Fran” workout (WOD_end_), and 15 min after its conclusion (Recovery). Heart rate was measured with a Polar RS800 device and a Polar chest strap (Polar Electro OY, Kempele, Finland). Age-predicted maximal heart rate was estimated according to Tanaka et al. [13].

According to the first aim of this study, body composition was first compared between CF participants who had been subdivided according to the amount of weekly CF training and then the relationship between body composition and performance of the “Fran” workout. Participants training for ≥ 10 hours a week over the course of the last year were the Higher-Training (HT, n = 13) group and those training for < 10 hours a week were the Lower-Training (LT, n = 11) group. Then, according to the second aim of the study, body composition of CF participants in each group (HT and LT) was compared with that of an age-matched (±6 y) and BMI-matched (±1 kg/m^2^) control group. According to recommendations for this type of study [14], control participants were physically active subjects extracted from a database including undergraduate and graduate kinesiology students as well as staff of the kinesiology departmental section accessing the sport facilities at the University. They all practiced sports (soccer, cycling, basketball, swimming, tracking etc.) at recreational level. Overall, four groups were created namely, the HT, control HT (CHT), LT, and control LT (CLT) group.

### Statistical analysis

Data were assessed for normality with the Shapiro-Wilk test. In the case of non-normal distribution, data were log_10_ transformed, resulting in normalized distribution. Between-group comparisons for anthropometry, body composition, performance, and blood variables were carried out with univariate One-way ANOVA in the General Linear Model according to the study design (HT vs. LT, HT vs. CHT, LT vs. CLT). Blood variables were compared at three time-points (Baseline, WOD_end_, Recovery) using repeated-measure ANOVA in the General Linear Model. Effect size was calculated as partial eta squared (η_p_^2^) and evaluated according to published reference [15] as small (0.01), medium (0.06) and large (0.14). Correlation between variables was assessed calculating Pearson’s r. Partial correlation analysis was also used where needed. The strength of correlation was rated as per Hopkins [16]: small (0–0.30), moderate (0.31–0.49), large (0.50–0.69), very large (0.70–0.89), and almost perfect (0.90–1). All analysis was performed with SPSS v. 25 (IBM Corp., Armonk, New York, USA). The alpha value was set at P = 0.05.

## Results

The characteristics of the two groups of CF participants are presented in Table 1.

**Table 1:** Characteristics of participants in the Higher Training (HT) and Lower Training (LT) groups. Data are means ± SD (Minimum-Maximum). One-way ANOVA.

Group	Age (y)	Body mass (kg)	Stature (cm)	CF experience (y)	Amount of training (h/w)
**HT (n = 13)**	28.5 ± 3.50 (22–35)	82.0 ± 5.93* (70.5–91.5)	175.9 ± 4.10 (170.1–182.7)	2.5 ± 0.75* (1.0–3.5)	12.0 ± 2.16* (10–18)
**LT (n = 11)**	27.9 ± 3.93 (23–36)	74.6 ± 6.17 (66.2–83.5)	176.9 ± 4.86 (172.2–186.9)	1.8 ± 0.93 (1.0–3.5)	7.3 ± 1.10 (6–9)

CF, CrossFit.

*, P < 0.05 vs. LT.

**Fig 1:**
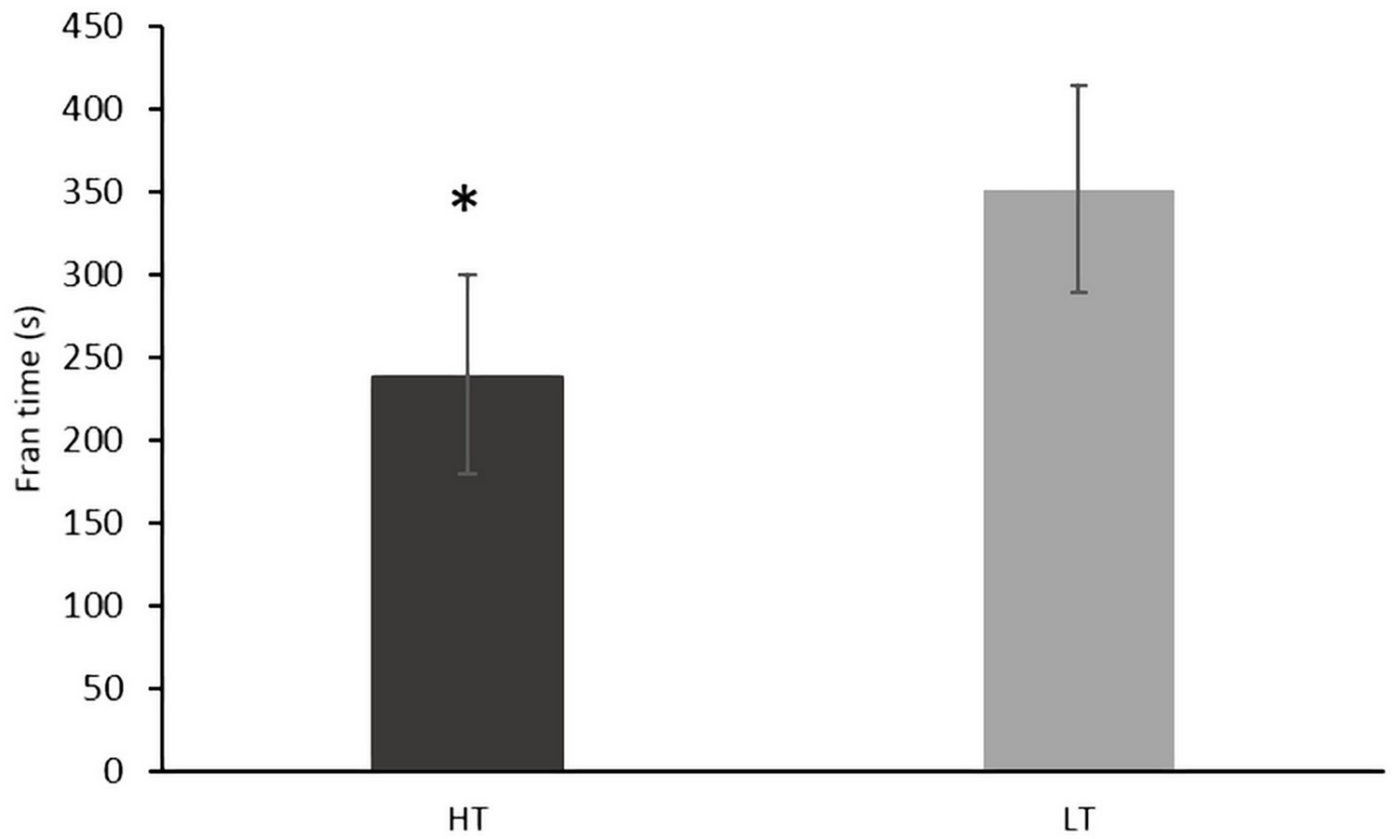
Performance in the “Fran” workout of the day in two groups of CrossFit participants. HT, higher training group; LT, lower training group. *, P < 0.05 vs. LT.

The HT and LT groups showed no statistically significant difference for age (F = 0.153, P = 0.700, η_p_^2^ = 0.007, power = 0.066) and stature (F = 0.260, P = 0.615, η_p_^2^ = 0.012, power = 0.078). The HT group showed higher body mass (F = 8.824, P = 0.007, η_p_^2^ = 0.286, power = 0.810) and longer CF experience (F = 3.091, P = 0.047, η_p_^2^ = 0.167). The amount of training was higher in HT vs. LT by design (P < 0.001). The HT group showed lower “Fran” time (F = 16.655, P < 0.001, η_p_^2^ = 0.431, power = 0.521; Fig 1). After including the amount of training or Appendicular LSTMI as a covariate in the model, the group effect respectively became borderline statistically significant (F = 3.696, P = 0.068, η_p_^2^ = 0.150, power = 0.450) and remained statistically significant (F = 4.982, P = 0.038, η_p_^2^ = 0.190, power = 0.563).

The HT and CHT group as well as the LT and CLT group had similar age (P = 0.926; P = 0.448, respectively) and BMI (P = 0.736; P = 0.805, respectively) by design.

### Body composition analysis

**Fig 2:**
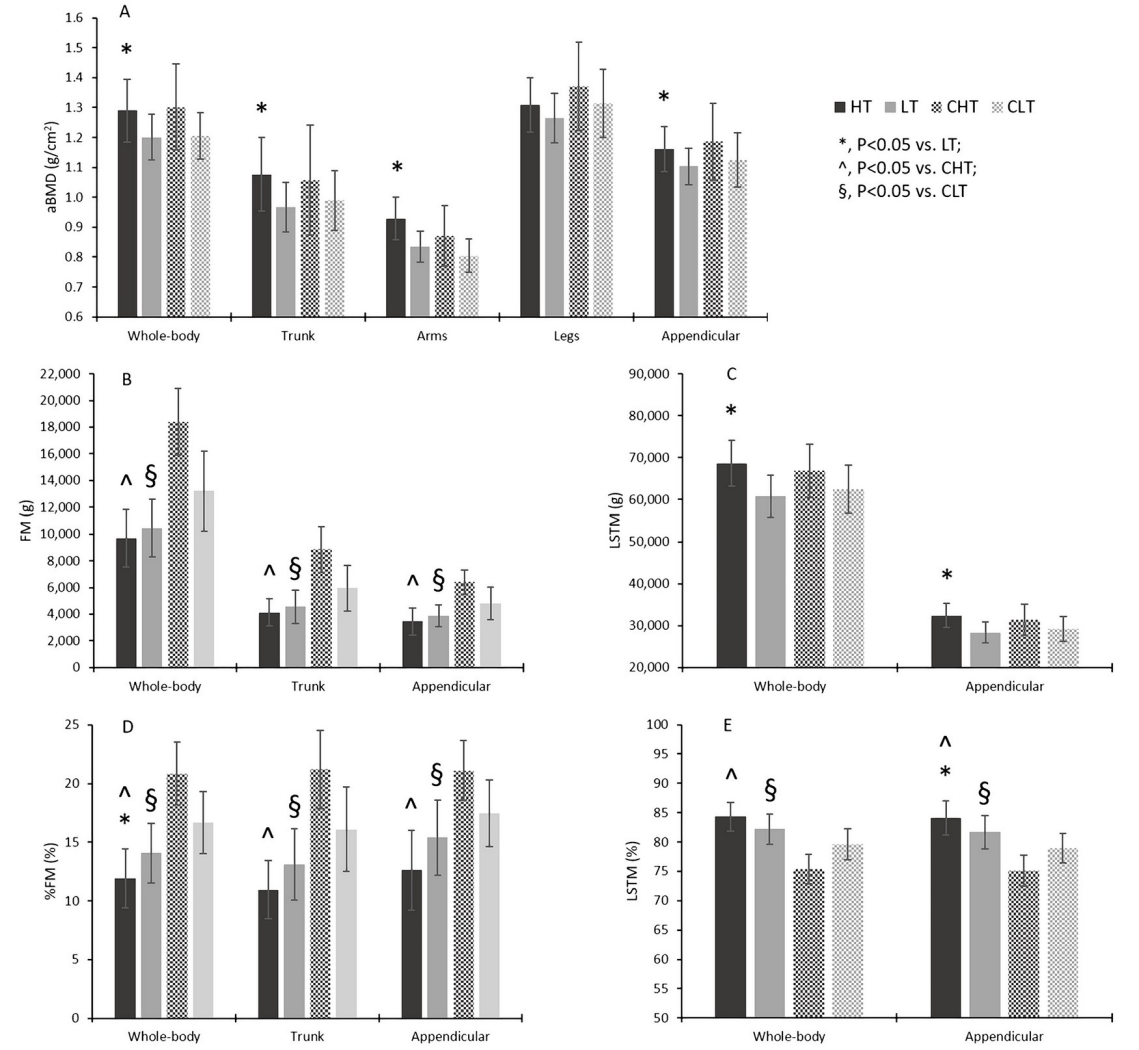
Whole-body and regional body composition in CF participants at higher (HT) and lower (LT) amounts of training, and in their age-matched and BMI-matched controls (CHT, CLT). aBMD, areal bone mineral density; FM, fat mass; LSTM, lean soft tissue mass.

Fig 2 shows aBMD, FM, and LSTM variables in the HT, LT, CHT, and CLT group. All mean absolute values of aBMD variables were higher in the HT vs. LT group (Fig 2, Panel A). However, One-way ANOVA showed statistically significant difference at the WB (F = 5.676, P = 0.026, η_p_^2^ = 0.205, power = 0.625), trunk (F = 6.251, P = 0.020, η_p_^2^ = 0.221, power = 0.666), Arms (F = 12.924, P = 0.002, η_p_^2^ = 0.370, power = 0.930), and Appendicular site (F = 4.346, P = 0.049, η_p_^2^ = 0.165, power = 0.513), but not at Legs (F = 0.231, P = 0.231, η_p_^2^ = 0.065, power = 0.218). After adjusting for CF experience, the difference remained significant for Arms aBMD (F = 7.640, P = 0.012, η_p_^2^ = 0.267, power = 0.750).

Participants in the HT group had lower mean FM and %FM values vs. LT (Fig 2, Panel B, D), the difference being statistically significant for WB %FM (F = 4.300, P = 0.050, η_p_^2^ = 0.164, power = 0.509) and, at the limit of statistical significance for the trunk %FM (F = 3.708, P = 0.067, η_p_^2^ = 0.144, power = 0.453). LSTM (Fig 2, Panel C) was higher in the HT group at both the WB and appendicular level (F = 13.040, P = 0.002, η_p_^2^ = 0.372, power = 0.932 and F = 13.504, P = 0.001, η_p_^2^ = 0.380, power, = 0.939, respectively). These differences remained statistically significant after adjusting for CF experience (F = 7.714, P = 0.011, η_p_^2^ = 0.269, power = 0.754 and F = 7.957, P = 0.010, η_p_^2^ = 0.275, power = 0.767, respectively). Percent LSTM (Fig 2, Panel E) was higher in the HT group, the difference being borderline statistically significant at WB (F = 4.141, P = 0.054, η_p_^2^ = 0.158, power = 0.494) and statistically significant at the Appendicular level (F = 4.387, P = 0.048, η_p_^2^ = 0.166, power = 0.517). After adjusting for CF experience, the latter difference became borderline statistically significant (F = 3.316, P = 0.083, η_p_^2^ = 0.136, power = 0.412).

Comparison of the HT and CHT groups (Fig 2) showed that WB and regional aBMD, and LSTM were not significantly different in the two groups. %LSTM was higher in HT at the WB- and appendicular levels (F = 86.085, P < 0.001, η_p_^2^ = 0.782, power = 1.000; F = 66.551, P <0.001, η_p_^2^ = 0.735, power = 1.000, respectively). The HT group had lower FM and %FM at the WB- and regional levels (F > 51.000, P < 0.001, η_p_^2^ > 650, power = 1.000 for all). Comparison of the LT and CLT group (Fig 2) showed non-statistically significant differences in aBMD and LSTM at the WB- and regional level. %LSTM was higher in LT at the WB- and Appendicular levels (F = 5.746, P = 0.026, η_p_^2^ = 0.223, power = 0.626; F = 5.550, P = 0.029, η_p_^2^ = 0.217, power = 0.611, respectively). The LT group had lower FM and %FM at the WB- and regional levels (F > 4.300, P ranging 0.050–0.020, η_p_^2^ > 0.180, power > 0.500).

For both LSTM and FM, the Appendicular value was only reported in Fig 2 because of consensual between-group changes in either the left and right arms or the left and right legs.

**Fig 3:**
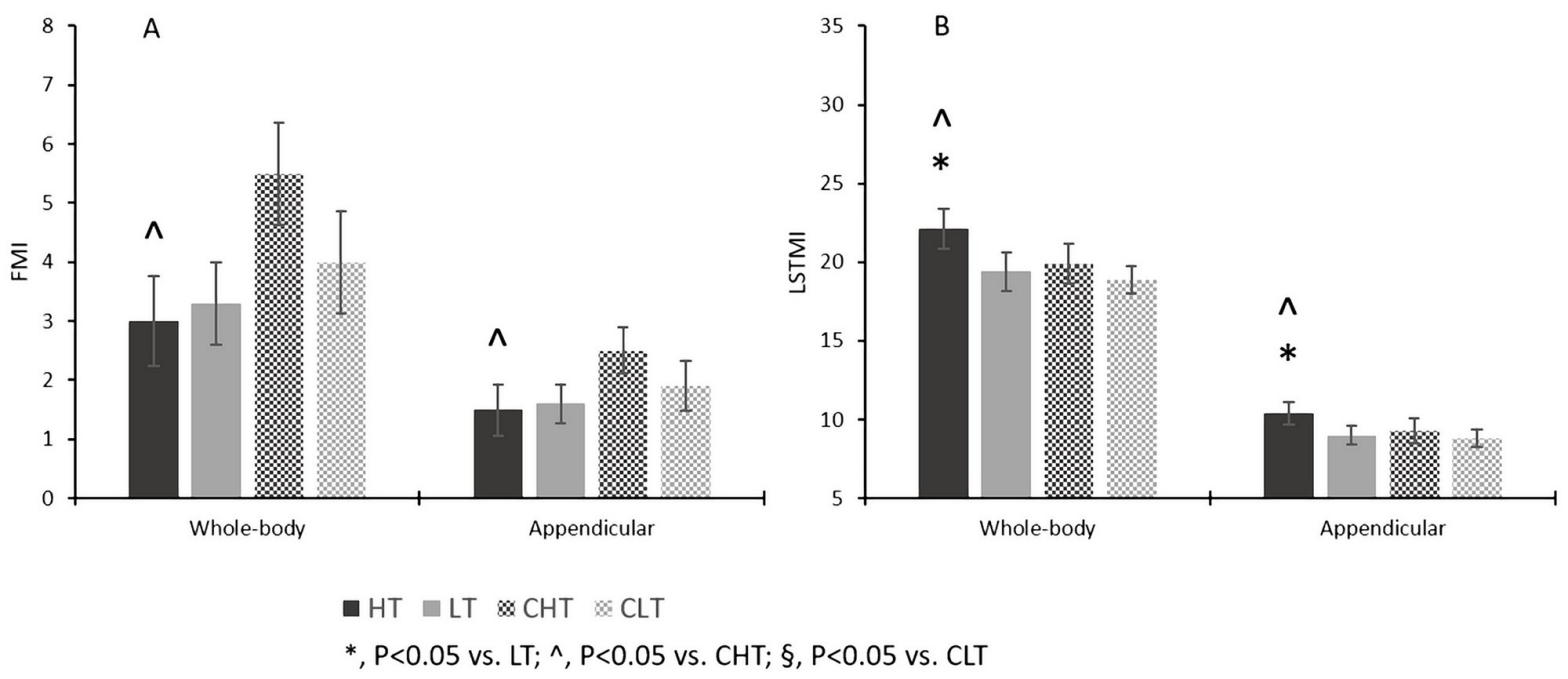
Whole-body and Appendicular Fat Mass Index (FMI, Panel A) and Lean Soft Tissue Mass Index (LSTMI, Panel B) in CF participants at higher (HT) and lower (LT) amounts of training, and in their age-matched and BMI-matched controls (CHT, CLT).

Fig 3 shows FMI and LSTMI in the four groups of study participants (HT, LT, CHT, CLT). WB FMI was similar in HT and LT group (F = 0.413, P = 0.527, η_p_^2^ = 0.018, power = 0.094). Similar results were found for Appendicular FMI. WB- and Appendicular LSTMI was higher in HT vs. LT (F = 28.705, P < 0.001, η_p_^2^ = 0.566, power = 0.999; F = 27.718, P < 0.001, η_p_^2^ = 0.558, power = 0.999, respectively) also after adjusting for CF experience (P < 0.001 for both). Considering the HT and CHT group, FMI was lower in HT (F = 54.736, P < 0.001, η_p_^2^ = 0.695, power = 1.000). LSTMI was higher in the HT group at the WB- level (F = 19.524, P < 0.001, η_p_^2^ = 0.449, power = 0.989) and appendicular level (F = 14.027, P = 0.001, η_p_^2^ = 0.369, power = 0.949). Considering the LT and CLT groups, WB FMI and Appendicular FMI were not statistically different (F = 3.861, P = 0.063, η_p_^2^ = 0.162, power = 0.464; F = 3.990, P = 0.060, η_p_^2^ = 0.166, power = 0.477, respectively). WB LSTMI and Appendicular LSTMI were not statistically different either (F = 1.320, P = 0.195, η_p_^2^ = 0.062, power = 0.264) (F = 0.630, P = 0.118, η_p_^2^ = 0.031, power = 0.437, respectively).

### Correlation analysis

**Fig 4:**
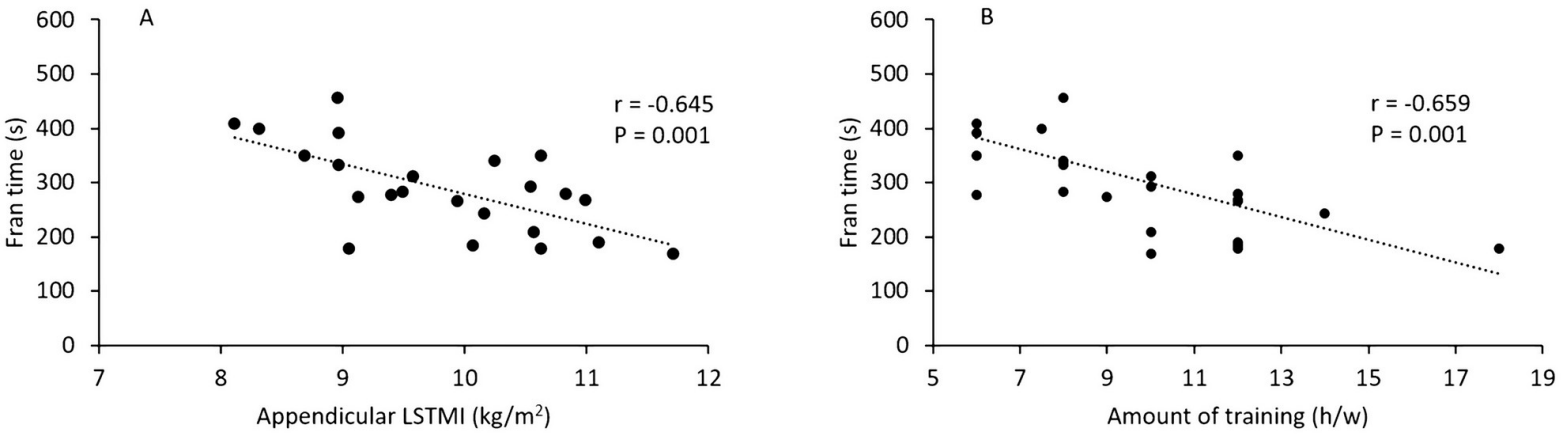
Association between performance in the “Fran” workout and either the amount of Appendicular Lean Soft Tissue Mass Index (LSTMI, Panel A) or the amount of weekly training (Panel B) in CF participants.

In the whole sample of CF participants (HT + LT), negative, statistically significant, moderate to large correlation was found between “Fran” time and both Appendicular LSTM (r = -0.454, P = 0.030) and Appendicular LSTMI (r = -0.645, P = 0.001; Fig 4A), but not between “Fran” time and WB FM (r = 0.124, P = 0.565), WB %FM (r = 0.322, P = 0.124) or WB FMI (r = 0.097, P = 0.654). The correlation between “Fran” time and Appendicular LSTMI remained statistically significant after adjusting for CF experience (r = -0.632, P = 0.002) and borderline statistically significant after adjusting for amount of training (r = -0.394, P = 0.070). Positive, moderate to very large correlations were found between aBMD variables and Appendicular LSTMI, which reached statistical significance for trunk aBMD (r = 0.443, P = 0.030), Arms aBMD (r = 0.742, P < 0.001), and Appendicular aBMD (r = 0.500, P = 0.013). After adjusting for CF experience, only the correlation between Arms aBMD and appendicular LSTMI remained significant (r = 0.549, P = 0.007). A negative large, statistically significant correlation was found between “Fran” time and weekly amount of training (r = -0.659, P = 0.001; Fig 4B). This correlation remained significant after adjusting for CF experience (r = -0.569, P = 0.005), body mass (r = -0.477, P = 0.021), stature (r = -0.583, P = 0.004), WB LSTM (r = -0.444, P = 0.034), and WB FM (r = -0574, P = 0.004).

### Capillary blood analysis

The results of lactate and glucose measurements are summarized in Table 2. Capillary blood lactate and capillary blood glucose at Baseline were in the normal range for resting people at fast (about 2.0 mmol/L and about 70 mg/dL, respectively).

**Table 2:** Capillary blood lactate and capillary blood glucose in the two groups of CF participants at rest (Baseline), at the end of the “Fran” workout (WOD_end_) and after a 15-min recovery (Recovery). Data are means ± SD. One-way ANOVA.

Group	Blood analysis	Baseline	WOD_end_	Recovery
HT (n = 13)	Lactate (mmol/L)	2.0 ± 0.91	14.8 ± 2.30	13.8 ± 2.22
	Glucose (mg/dL)	69.4 ± 13.78	90.8 ± 31.06	97.9 ± 23.36
LT (n = 11)	Lactate (mmol/L)	2.0 ± 1.02	14.6 ± 2.37	12.8 ± 1.97
	Glucose (mg/dL)	74.3 ± 17.04	97.4 ± 27.14	108.8 ± 24.12

HT, Higher Training; LT, Lower Training.

The HT and LT groups showed similar capillary blood lactate and capillary blood glucose at Baseline (F = 0.000, P = 0.987; F = 0.604, P = 0.445, respectively). On average, capillary blood lactate markedly increased from rest (Baseline) to the end of the “Fran” workout (WOD_end_) in both groups (HT, + 740%; LT, + 730%) and moderately decreased from WOD_end_ to Recovery (HT, -6.8%; LT, -12.0%). Capillary blood glucose showed a moderate increase from Baseline to WOD_end_ in both groups (HT, + 30.8%; LT, + 31.1%) and increased even further from Baseline to Recovery (HT, + 41.1%; LT, + 46.4%). Repeated-measure ANOVA showed no significant interaction between group and metabolite pattern for either capillary blood lactate (F = 0.448, P = 0.642, η_p_^2^ = 0.020, power = 0.118) or capillary blood glucose (F = 0.215, P = 0.807, η_p_^2^ = 0.010, power = 0.082). Average heart rate during the “Fran” workout was 92.7 ± 5.38% and 94.06 ± 3.72% of age-predicted maximal heart rate in the HT and LT groups, with a maximum of 97.4 ± 5.31% and 98.3 ± 3.65%, respectively. Both differences were not statistically significant (F = 0.460, P = 0.505; η_p_^2^ = 0.020, power = 0.099 and F = 0.220, P = 0.644, η_p_^2^ = 0.010, power = 0.073, respectively).

## Discussion

The first aim of this study was to assess the effect of different amounts of HIFT training on body composition and its association with CF performance. Results showed a statistically significant main effect of the amount of training (12 h/w on the average in HT; 7 h/w on the average in LT; duration of at least 1 y) on several body composition variables as well as a statistically significant, positive association of Appendicular LSTM (a proxy of skeletal muscle mass) with performance of the “Fran” WOD.

Results in CF participants came from subjects with a sustained, continuative participation in HIFT (1–3.5 years of CF participation; 6–18 h/w of training). Therefore, findings should be considered representative of a maintenance stage and reflect stable training status and CF ability. To the best of our knowledge, this is the first study investigating CF athletes at “steady state” body composition. Previous work [17–19,10] investigated changes in body composition on a reduced timespan (5–16 w), which may not have led to a steady state.

Comparison of aBMD in HT and LT showed that higher weekly amount of training is associated with higher aBMD at all sites but Legs, with a large effect size. Accordingly, it could be envisaged that a higher amount of CF training is beneficial to bone health. Nevertheless, the HT group showed statistically significant longer CF experience (Table 1). After controlling for CF experience, a significant difference persisted between HT and LT for Arms aBMD only (P = 0.012); this indicates that cumulative exposure to impact training exercise was more effective than amount of training on aBMD in the weight-bearing skeleton. Actually, Arms aBMD was 11.9% higher in HT vs. LT. Taking into account that a 5.4% increase in aBMD is equal to a 64% increase in ultimate force and 94% increase in energy to failure in experimental animals [20], it is suggested that higher amount of CF training had an important effect on bone health of non-weight bearing parts of the skeleton, which was independent of body mass and duration of CF participation. It is well known that when bone is mechanically loaded, a response will occur in that specific bone [21–24]. Accordingly, a higher amount of CF training could have led to differential mineralization of upper limb bones due to a high mechanical load on the arms through several multicompartment exercises such as thrusters, push-ups, wall balls, clean, deadlift and kettle-bell swings, all of which are currently performed in CF practice. Interestingly, a statistically significant relationship was found between Arms aBMD and Appendicular LSTMI, which remained significant even after adjusting for CF experience. LSTMI has been positively associated with aBMD at the WB- and regional levels in young men [25,26], indicating that increasing skeletal muscle mass may contribute to increased aBMD at the upper limb. The hypothesis that the non-weight bearing parts of the skeleton respond to CF participation in a dose-dependent manner deserves further investigation.

In our sample of CF participants, WB LSTM was higher in HT vs. LT (+12.8%, P = 0.002) with a large effect size; such an effect was independent of CF experience i.e., the cumulative amount of previous training, which was on the average 39% longer in HT. WB %LSTM was also higher in HT (+2.1%) at the limit of statistical significance (P = 0.054) with a large effect size. More interestingly, Appendicular LSTM (a proxy of body skeletal muscle) was higher in HT (+14.2%, P = 0.001) independent of CF experience (P = 0.010) and Appendicular %LSTM was higher in HT as well (+2.4, P = 0.048). Overall, these findings indicate that a higher amount of training benefits the body’s lean mass as a whole in CF participants, especially by enhancing skeletal muscle accrual. As shown by LSTMI analysis (Fig 3), this effect was independent of stature, the major determinant of body lean mass. Interestingly, statistically significant, positive correlations were found between Appendicular LSTM and Appendicular LSTMI, and performance of the “Fran” WOD (P = 0.030; P = 0.001, respectively) suggesting that increasing amounts of skeletal muscle lead to better performance. When interpreting these results, it should however be kept in mind that skeletal muscle mass and strength may not parallel in individuals [27]. We did not assess muscle function in this work and, therefore, cannot comment on possible differences in muscle quality between CF participants training at different weekly amounts. Indirect evidence that better muscle function was present in the HT group comes from the discovery that the difference between HT and LT in “Fran” time (P < 0.001) remained significant after adjusting for Appendicular LSTMI (P = 0.038) and became borderline significant (P = 0.068) after adjusting for weekly amount of training.

In our sample of CF participants (HT and LT groups combined) %FM ranged 8.6–16.9. This range includes values at or below the 50^th^ percentile in different athletic populations [28], showing that CF participation (≥ one year) was effective in achieving excellent fitness. Comparison of the HT and LT group showed that different weekly amount of training has limited impact on the fat component of body composition, the difference in both FM and FMI being not statistically different and WB %FM being lower in HT at the limit of statistical significance (P = 0.050). These findings indicate that higher amounts of training over a sustained (≥ one year) period did not convey substantial additional benefits to participants in terms of body adiposity.

In this work we assessed the relationship between body composition and CF performance using a typical CF WOD (the “Fran”). The average score of HT participants (Fig 1) was superimposable to that obtained by a sample of male CF participants (237.9 ± 83.9 s; n = 12; age = 29.0 ± 5.6; CF experience = 49.0 ± 32.7 months; [29]). Normative data collected on CF benchmark workout performance [30] indicate that participants in the present study ranked between the 50^th^ and 60^th^ percentile (HT group), and the 10^th^ and 20^th^ percentile (LT group). Accordingly, CF participants in this study were representative of recreational (LT) or intermediate (HT) CF practitioners.

Correlation analysis showed no association between “Fran” time and FM, %FM, and FMI showing that body adiposity was not a factor in performance. Instead, “Fran” time improved with increasing LSTMI, this association showing large strength (r = -0.645, P = 0.001). The statistical significance of such an association was almost identical after adjusting for CF experience but lost statistically significance after adjusting for the amount of training, indicating that the intensity of CF participation benefits the utilization of skeletal muscle to a greater degree than a longer exposure to CF practice. Accordingly, a statistically significant negative correlation was found between the weekly amount of training and the time employed to carry out the “Fran” WOD (P = 0.003), which was independent of anthropometry, body composition, and CF experience. Overall, results showed that a positive relationship exists between the amount of training and the performance in CF, similar to that found in other sports settings [31–33].

During the “Fran” WOD, average heart rate was > 92% maximal estimated heart rate in both HT and LT, indicating that participants were performing at high intensity. According to repeated-measures ANOVA, the patterns of capillary blood lactate and capillary blood glucose at baseline, at the end of the “Fran” WOD and after 15 min of recovery were similar in the HT and LT group. Capillary blood lactate showed a sharp increase at the end of the “Fran” WOD similar to that attained in similar bouts of HIFT [34–37], which was followed by a moderate decline during recovery. According to findings in previous studies [34,38] a steeper decrease in blood lactate was expected. While differences in the metabolic requirement of HIFT exercises could explain such a discrepancy, investigation of lactate kinetics is to be carried out to clarify lactate clearance after the “Fran” WOD. Capillary blood glucose showed a moderate increase at the end of the “Fran” WOD followed by a further, modest increase after the 15 min recovery period. This is consistent with the pattern of blood glucose found in endurance athletes after intense exercise [39,40]. During intense exercise, glucose production rises seven- to eightfold with glucose utilization rising three- to fourfold and, therefore, glycemia increases; at the end of exercise, glucose utilization initially decreases more than glucose production, leading to greater hyperglycemia [41]. The lack of a statistically significant difference in the pattern of capillary lactate and capillary glucose in the HT and LT groups over the course of the “Fran” WOD shows that different amounts of training did not induce relevant modulation of the metabolic response to an HIFT challenge in CF participants.

The second aim of this work was to assess whether CF participation is associated with differences in body composition vs. age-matched and BMI-matched physically active controls. Results partially confirmed this hypothesis by showing several statistically significant differences in FM but not in LSTM or aBMD for the HT and LT groups vs. their respective controls.

HT and LT did not have statistically different WB- and regional aBMD vs. CHT and CLT, respectively, with small between-group mean differences and small to medium effect size. This is consistent with similar findings of mean WB- and regional aBMD in nine recreationally active men (34.2 ± 9.1 y; 91.5 ± 17.7 kg; 178.5 ± 5.4 cm) after a 16-week HIFT participation (minimum twice a week; [10]). Accordingly, it is suggested that CF participation for at least one year is generally not associated with better bone characteristics vs. age-matched and BMI-matched controls. However, a high percentage difference between mean aBMD values was found in Arms aBMD (HT vs. CHT, +6.6% and LT vs. CLT, +3.7%). The effect size was large for HT vs. CHT (η_p_^2^ = 0.370) and moderate for LT vs. CLT (η_p_^2^ = 0.075), indicating that CF participation may have a positive effect on upper limb bone mineralization at both higher and lower amounts of training.

Appendicular LSTM was not statistically different in the HT or LT groups and their respective controls, suggesting that CF participation had no major effect on the absolute amount of skeletal muscle mass at similar age and BMI irrespective of the amount of weekly training. However, normalizing data for body mass (%LSTM) or stature (LSTMI) revealed statistically significant or borderline statistically significant differences between both the HT and LT groups, and their respective controls (Fig 2, Panel C and E; Fig 3, Panel A) showing that CF participation might have a positive impact on relative body lean soft tissue mass. Once again, it should be mentioned that we did not perform muscle function testing in this study and, consequently cannot comment on possible effects of CF participation on muscle quality vs. control.

HT and LT had statistically significant or borderline statistically significant lower WB and regional FM, %FM, and FMI vs. CHT and CLT, respectively, (Fig 2, Panel B and D; Fig 3, Panel A) the effect size being large for all comparisons (η_p_^2^, 0.162–0.792). Accordingly, CF participation for at least one year led to an improvement of absolute and relative body adiposity vs. age-matched and BMI-matched controls. It was found that between-group mean differences are markedly larger in HT vs. CHT than LT vs. CLT. This is explained by the inclusion of several participants with BMI > 25 kg/m^2^ in the CHT group, in order to match HT participants for BMI. Despite being physically active, these controls had higher adiposity than those in the CLT group, hence the larger mean difference of FM and %FM in HT vs. CHT than LT vs. CLT.

In this work, CF participants and controls were matched for BMI according to current practice in sports science [42–45] especially when bone-related variables are to be compared. This may have affected results to some extent as specified in the paragraph above. In order to put the current results on body composition in CF participants into a more complete perspective, comparison is made in the following to the reference general population.

In our sample of CF participants, the range of %FM (8.6%– 16.9%) was well below the mean DXA-measured value for a reference population (20.3% for age range 18–24 y; 25,6% for age range 25–34 y; [46], thereby confirming that CF exposure has a positive effect on the participant’s fitness. It should also be considered that HT had higher BMI vs. LT (+1.7 kg/m^2^, P < 0.001) with 11 out of 13 HT subjects and two out of 11 LT participants showing BMI > 25 kg/m^2^ i.e., the current cut-off for overweight (WHO, [47]). However, it is well known that BMI does not take into account body composition and, accordingly, a man with an overweight BMI can have an infinite variety of FM to lean mass ratios i.e., he may have only a little FM and a large amount of lean mass [48–50]. In our sample of CF participants, individuals with BMI > 25 kg/m^2^ presented higher proportions of lean mass. As far as lean mass is concerned, comparison of WB LSTM with DXA-measured values available for a male Italian population [46], showed that an average value in HT (68.6 kg) and LT (60.8 kg) was close to the 90^th^ percentile (67.46 kg for age range 18–24 y; 73.10 kg for age range 25–34 y) for the former and between the 50^th^ (56.90 for age range 18–24 y and 58.00 for age range 25–34 y) and 75^th^ percentile (62.70 for age range 18–24 y and 64.80 for age range 125–34 y) for the latter. In comparison with DXA measurements available for an Italian population [51], Appendicular LSTM in HT (32.37 kg) was near the 95^th^ percentile (35.32 kg for age range 20–29 y; 34.31 kg for age range 30–39). In LT, Appendicular LSTM (28.35 kg) was close to the 50^th^ percentile (28.30 kg for age range 20–29 y and 27.75 kg for age range 30–39). Since LSTM is closely related to stature [50], it could be argued that differences in this anthropometric variable affected result. Instead, analysis of Appendicular LSTMI (kg/m^2^) confirmed LSTM results by showing values in HT and LT (10.4 kg/m^2^ and 9.0 kg/m^2^, respectively) which were close to the 95^th^ percentile (10.33 kg/m^2^ for age range 20–29 y; 11.23 kg/m^2^ for age range 30–39 y) and the 50^th^ percentile (8.74 kg/m^2^ for age range 20–29 y; 8.78 kg/m^2^ for age range 30–39 y) of a reference population [51], respectively. Therefore, CF participation with a weekly amount of training < 10 h/w for at least one year (mean participation time, 1.8 y) did not lead to skeletal muscle mass accrual beyond that of the general population.

There are some limitations of this study that should be mentioned. First, only 24 young adult male athletes were recruited, which limits the ability to generalize results to the entire population of CF participants. Further research is needed to explore whether the current results can be extended to other populations of CF athletes such as females and younger athletes. Second, we were not able to collect precise information on the dose and type of training carried out by participants or their dietary regimen, which could affect their body composition. Third, we only assessed CF performance with the “Fran” WOD. Future studies will need to explore the relationship between the amount of training, body composition and performance by including other WODs.

## Conclusions

To best of our knowledge this is the first study investigating the impact of different amounts of training on body composition and performance of CF participants. Overall, our findings showed that in athletes with CF experience > 1y, a higher amount of weekly training (> 10 h/w) is associated with better body composition, especially in terms of lean mass and upper limb bone density, as well as better performance in a typical CF workout. This is of importance in athletic performance, providing CF coaches withuseful information to define the amount of weekly training necessary to enable positive effects on body composition and performance. Another finding of this study was that CF participation ≥ 6 h/w determines a better body composition profile (i.e. higher lean mass and lower FM) in comparison with age-matched and BMI-matched physically active controls. This could help fitness trainers using CF as a fitness strategy in prescribing suitable amounts of training able to improve body composition. Further investigation of the relationships between CF participation, body composition and anaerobic power and capacity as well as functional strength will give better insight into the determinants of CF performance. Given the increasing usage of CF as a training modality in different sport and fitness settings, research is also needed to further explore the effects of CF on specific health and performance outcomes.

## Supporting information

S1 File Database. (CSV)
